# Visceral adiposity rather than BMI predicts overall survival after curative gastrectomy for gastric cancer: a retrospective cohort study with age-stratified analyses

**DOI:** 10.3389/fmed.2026.1792163

**Published:** 2026-03-18

**Authors:** Yafei Liu, Dong Hou, Tao Meng, Xiangjie Fang, Yong Tan, Xiaoyang Li

**Affiliations:** Department of General Surgery, The First Affiliated Hospital of Henan Medical University, Weihui City, Henan Province, China

**Keywords:** body mass index, curative gastrectomy, gastric cancer, obesity paradox, visceral fat area

## Abstract

**Objective:**

The prognostic value of body mass index (BMI) remains contested in gastric cancer, in part because BMI conflates distinct compartments of body composition. This study examined the respective associations of BMI and computed tomography–derived visceral fat area (VFA) with postoperative morbidity and overall survival (OS) after curative gastrectomy, while also probing whether age modifies these relationships.

**Methods:**

A retrospective cohort was assembled from patients undergoing curative-intent gastrectomy for gastric cancer at the First Affiliated Hospital of Henan Medical University between 2015 and 2019. BMI was grouped using WHO Asia–Pacific thresholds, whereas VFA was quantified on preoperative CT images at the L3 level. OS was prespecified as the primary endpoint, with postoperative complications treated as a secondary endpoint. Survival was characterized using Kaplan–Meier estimation and tested with Cox proportional hazards modeling under multivariable adjustment. Age-stratified analyses were implemented to evaluate effect modification.

**Results:**

Among 868 eligible patients, underweight status was associated with significantly worse OS, whereas higher BMI categories were not accompanied by an increased mortality risk. In contrast, elevated VFA independently predicted inferior OS irrespective of BMI. The BMI–OS association differed across age strata, suggesting age-dependent heterogeneity in the prognostic meaning of BMI, while the adverse association between visceral adiposity and survival was comparatively consistent across age groups. Correlation between BMI and VFA was weak, reinforcing the premise that these metrics encode non-overlapping biological information.

**Conclusion:**

In gastric cancer patients treated with curative gastrectomy, BMI and fat distribution appear to convey divergent prognostic signals. Visceral adiposity, as indexed by CT-derived VFA, provides survival-related information beyond BMI and may more effectively flag patients at heightened risk for adverse outcomes. Routine incorporation of CT-based body composition profiling into preoperative evaluation could refine risk stratification and support more individualized perioperative management.

## Introduction

Gastric cancer remains a leading cause of cancer-related death worldwide, with a particularly high burden in East Asia. Although curative gastrectomy offers the best chance of long-term disease control for resectable tumors, postoperative morbidity and survival vary widely across patients even under standardized perioperative pathways (1, 2). Identifying host-related, actionable predictors that can be integrated into preoperative risk assessment therefore remains clinically important (3).

Body size and nutritional status are frequently discussed prognostic factors in surgical oncology (4, 5). Most studies have used body mass index (BMI) as the default proxy for obesity, yet BMI-based associations with outcomes after gastrectomy are inconsistent and sometimes compatible with an “obesity paradox,” in which underweight patients fare poorly while overweight/obese patients do not show worse survival (6–8).

A key limitation is that BMI conflates distinct components of body composition and ignores fat distribution (9). In addition, BMI cut-offs used to define overweight/obesity differ between East Asian and Western populations (e.g., Asia–Pacific thresholds vs. the conventional Western obesity threshold of ≥30 kg/m^2^), which complicates cross-study comparisons and limits BMI’s ability to reflect visceral adiposity and muscle reserve across settings.

Visceral adipose tissue is metabolically active and more closely linked than subcutaneous fat to insulin resistance, chronic low-grade inflammation, and adipokine dysregulation. These pathways plausibly influence tumor biology, host immunity, and treatment tolerance. From a surgical standpoint, excess visceral fat may also increase technical difficulty during gastrectomy, potentially affecting perioperative outcomes (10, 11).

Computed tomography (CT), already part of routine staging and operative planning, permits quantitative assessment of visceral fat area (VFA) without additional patient burden (12). Prior studies have associated high VFA with greater operative difficulty and, in some cohorts, increased complications. However, evidence on long-term prognosis is mixed. Some reports link visceral adiposity to inferior survival, whereas others suggest neutral or paradoxical associations, indicating that population characteristics, tumor stage, and analytic definitions may shape observed relationships (13).

Age further complicates interpretation because aging is accompanied by skeletal muscle loss and preferential fat redistribution toward visceral depots, sometimes with minimal BMI change (14). Thus, the prognostic meaning of a given BMI may differ between younger and older patients. Whether age modifies the associations of BMI and CT-derived visceral adiposity with outcomes after curative gastrectomy remains insufficiently studied.

Against this backdrop, we conducted a retrospective cohort study of gastric cancer patients undergoing curative gastrectomy to compare associations of BMI and CT-quantified VFA with postoperative complications and overall survival (OS), and to evaluate effect modification by age.

## Materials and methods

### Study design, setting, and ethics

A retrospective cohort design was adopted at the First Affiliated Hospital of Henan Medical University. Medical records of patients treated for gastric cancer between January 2015 and December 2019 were reviewed. The protocol received approval from the institutional Ethics Committee, and study conduct adhered to the Declaration of Helsinki and relevant local regulatory requirements.

### Participants and eligibility criteria

Eligible participants were adults (≥18 years) with histopathologically confirmed gastric adenocarcinoma who underwent curative-intent radical gastrectomy during the study window. Inclusion was restricted to patients achieving R0 resection, defined as the absence of microscopic residual tumor. Exclusion criteria comprised: missing or incomplete data required to derive BMI or quantify VFA, evidence of metastatic disease at the time of surgery, and absence of follow-up information. After application of these criteria, 868 patients constituted the analytic cohort.

### Data sources and variable ascertainment

Demographic characteristics (age, sex) and perioperative clinical information were extracted from the electronic medical record system. Preoperative height and weight were recorded as part of routine assessment, and BMI was calculated as weight (kg) divided by height squared (m^2^). BMI categories were defined using an Asia-Pacific/WHO approach as underweight (<18.5 kg/m^2^), normal (18.5–22.9 kg/m^2^), overweight (23.0–24.9 kg/m^2^), and obese (≥25.0 kg/m^2^).

Visceral adiposity was quantified on preoperative abdominal CT imaging. VFA was measured on a single axial slice at the level of the third lumbar vertebra (L3) using validated body-composition software and expressed in square centimeters. CT was obtained as part of routine preoperative staging; VFA was assessed only on the preoperative scan (no longitudinal or postoperative body-composition measurements were performed). In this cohort, preoperative CT was performed within 30 days before surgery. Visceral obesity was defined *a priori* as VFA ≥ 100 cm^2^, consistent with commonly applied thresholds in gastric cancer imaging studies. Patients were classified as low VFA (<100 cm^2^) or high VFA (≥100 cm^2^).

Clinical covariates included comorbidities, operative characteristics, and pathologic features. Diabetes mellitus, hypertension, chronic obstructive pulmonary disease, and coronary heart disease were coded as present when documented in the medical history. Surgical variables (extent/type of gastrectomy and lymphadenectomy) and tumor characteristics (pathologic stage and nodal status) were abstracted from operative and pathology reports. Perioperative systemic therapy was recorded, including neoadjuvant chemotherapy and adjuvant chemotherapy.

All patients underwent preoperative nutritional risk screening using the Nutritional Risk Screening 2002 (NRS 2002) tool. Routine prophylactic preoperative enteral access (e.g., feeding jejunostomy, nasogastric/nasojejunal feeding tubes) was not used in this cohort.

### Postoperative complications

Complications occurring within 30 days after surgery were recorded and graded using the Clavien–Dindo classification. Major complications were defined as grade III or higher, reflecting events requiring surgical/endoscopic/radiologic intervention or associated with life-threatening organ dysfunction.

### Outcomes and follow-up

OS served as the primary endpoint and was defined as the interval from the date of surgery to death from any cause; patients alive at last contact were censored at that date. Postoperative morbidity was treated as the secondary endpoint, with particular emphasis on major complications (Clavien–Dindo ≥III). Follow-up information was obtained through scheduled outpatient visits, structured telephone contact, and review of institutional records (including mortality registries where available). The administrative censoring date was December 2021. Patients without verifiable follow-up were classified as lost to follow-up and were excluded as specified above.

### Statistical analysis

Analyses were performed with patients grouped by BMI category (underweight, normal, overweight, obese) and by visceral fat category (low vs. high VFA). Categorical variables were summarized as counts and percentages. Continuous variables were summarized as medians with interquartile ranges (IQR), reflecting non-normal distributions typical of clinical measurements unless otherwise indicated.

Between-group comparisons for categorical variables were conducted using the chi-square test; Fisher’s exact test was applied when expected cell counts were <5. Continuous variables were compared using Student’s t-test when normality assumptions were satisfied; otherwise, the Mann–Whitney U test was used.

Survival functions were estimated by the Kaplan–Meier method and compared with the log-rank test. Cox proportional hazards regression was then used to quantify associations with OS. Candidate covariates included age, sex, BMI category, VFA group, Stage I/II/III, receipt of adjuvant chemotherapy, and comorbidity burden. Univariable Cox models were fitted first. Variables demonstrating statistical association in univariable analyses (*p* < 0.05), as well as variables considered clinically relevant *a priori*, were entered into multivariable models. Hazard ratios (HRs) with 95% confidence intervals (CIs) were reported.

To probe potential age-related effect modification, subgroup analyses stratified by age (<60 vs. ≥60 years) were conducted. Interaction terms between age group and BMI (or VFA) were evaluated within Cox models to test whether adiposity–survival associations differed across age strata.

All tests were two-sided, with *p* < 0.05 denoting statistical significance. Analyses were conducted in R (R Foundation for Statistical Computing, Vienna, Austria), version 4.5.2. Reporting followed STROBE recommendations for observational studies.

## Results

### Patient characteristics

In total, 868 patients with gastric cancer were analyzed. On the basis of BMI, 13.4% were classified as underweight (BMI < 18.5 kg/m^2^; *n* = 116), 37.3% as normal weight (18.5–22.9 kg/m^2^; *n* = 324), 13.5% as overweight (23.0–24.9 kg/m^2^; *n* = 117), and 35.8% as obese (≥25.0 kg/m^2^; *n* = 311). Mean age clustered around 60 years across the BMI strata. Although the cohort was predominantly male overall (73.3%), sex distribution was not uniform: the underweight subgroup exhibited a comparatively higher proportion of females, with only 54.3% male, whereas the higher-BMI categories were largely male (75.2%).

Despite these BMI differences, VFA showed little separation at the group level, averaging approximately 95–103 cm^2^ in each category. Tumor stage was likewise broadly comparable across strata, with Stage I representing roughly 23–24%, Stage II ~ 48–50%, and Stage III ~ 25–33% in most BMI groups; the obese subgroup appeared to concentrate more heavily in Stage II (64.1%) with fewer Stage III cases (12.5%). With respect to baseline comorbidity, hypertension (~32%) and coronary disease (~3–6%) were distributed with minimal variation by BMI. Diabetes mellitus was documented in 29/116 (25.0%) of underweight patients, 79/324 (24.4%) of normal-weight patients, 26/117 (22.2%) of overweight patients, and 62/311 (19.9%) of obese patients.

Use of systemic therapies was generally consistent across BMI strata. Approximately 30% of patients received neoadjuvant or adjuvant chemotherapy, while immunotherapy exposure ranged from ~5 to 12% without an obvious BMI-dependent gradient. Surgical management followed a similarly parallel distribution, with about one-third of patients in each BMI group undergoing total gastrectomy (Table 1).

**Table 1:** Baseline patient characteristics by BMI category (*N* = 868).

Characteristic	Underweight (BMI < 18.5) (*n* = 116)	Normal (BMI 18.5–22.9) (*n* = 324)	Overweight (BMI 23.0–24.9) (*n* = 117)	Obese (BMI ≥ 25.0) (*n* = 311)
Age, years (mean ± SD)	60.0 ± 13.8	60.0 ± 15.1	58.4 ± 14.3	59.3 ± 14.6
Visceral fat area, cm^2^ (mean ± SD)	95.6 ± 17.2	100.2 ± 16.6	105.6 ± 15.7	100.9 ± 16.2
Male sex, *n* (%)	63 (54.3%)	257 (79.3%)	97 (82.9%)	234 (75.2%)
Tumor stage				
– Stage I, *n* (%)	26 (22.4%)	74 (22.8%)	31 (26.5%)	74 (23.8%)
– Stage II, *n* (%)	51 (44.0%)	153 (47.2%)	60 (51.3%)	165 (53.1%)
– Stage III, *n* (%)	39 (33.6%)	97 (29.9%)	26 (22.2%)	72 (23.2%)
Treatment received				
– Neoadjuvant/adjuvant chemotherapy, *n* (%)	35 (30.2%)	107 (33.0%)	31 (26.5%)	91 (29.3%)
– Immunotherapy, *n* (%)	14 (12.1%)	26 (8.0%)	12 (10.3%)	25 (8.0%)
Comorbid conditions				
– Diabetes mellitus, *n* (%)	29 (25.0%)	79 (24.4%)	26 (22.2%)	62 (19.9%)
– Hypertension, *n* (%)	35 (30.2%)	111 (34.3%)	35 (29.9%)	101 (32.5%)
– Coronary heart disease, *n* (%)	7 (6.0%)	11 (3.4%)	3 (2.6%)	16 (5.1%)
– COPD (chronic lung disease), *n* (%)	6 (5.2%)	21 (6.5%)	8 (6.8%)	14 (4.5%)
Surgery type				
– Total gastrectomy, *n* (%)	46 (39.7%)	105 (32.4%)	39 (33.3%)	115 (37.0%)

BMI, body mass index; VFA, visceral fat area. BMI categories followed the WHO Asia–Pacific recommendations: Underweight (<18.5 kg/m^2^), Normal (18.5–22.9), Overweight (23.0–24.9), and Obese (≥25.0). Values are presented as mean ± standard deviation for continuous variables and as number (%) for categorical variables.

### Survival analysis by BMI category

**Figure 1:**
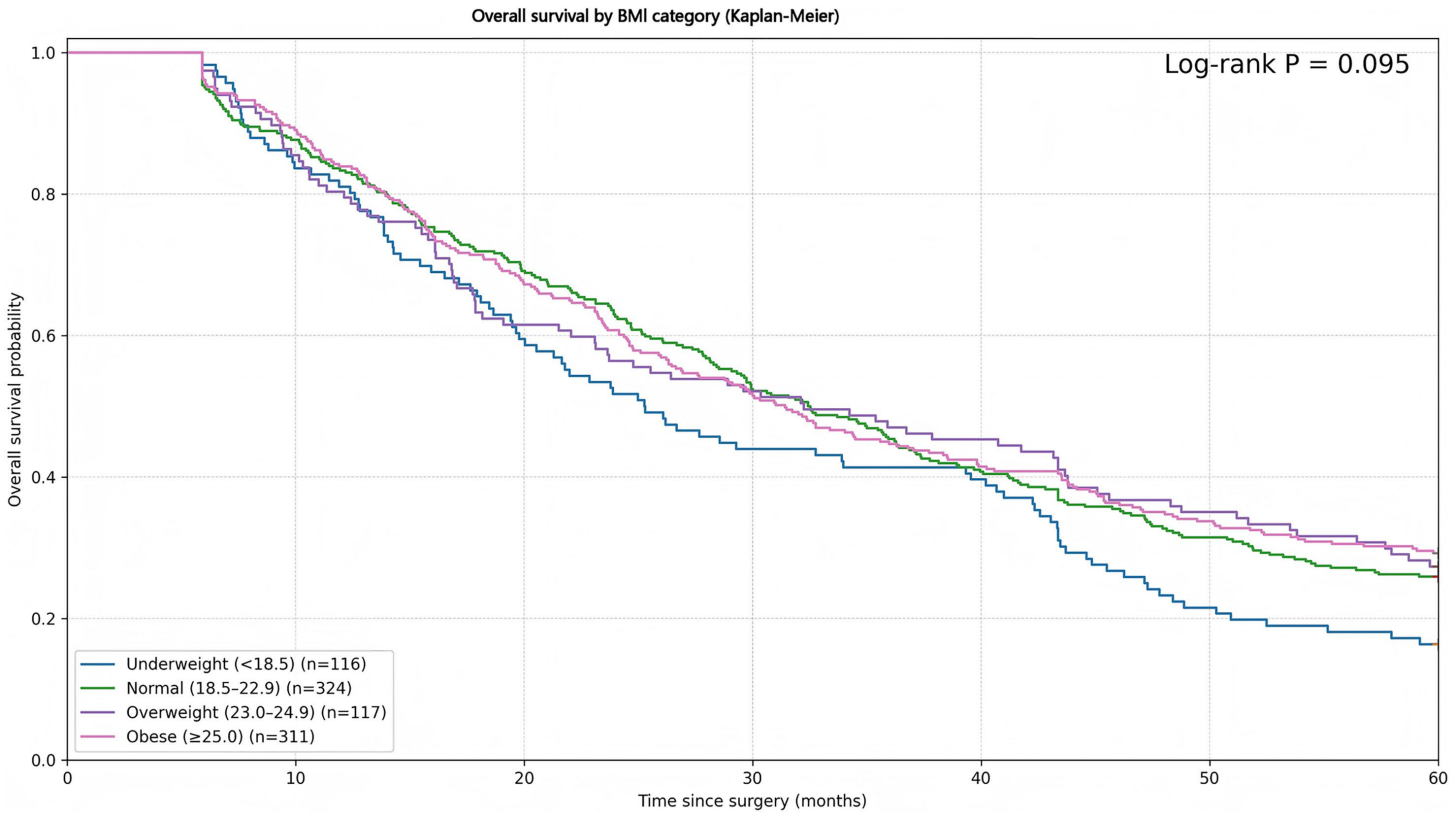
Overall survival (OS) by BMI category. Kaplan–Meier curves for OS after curative gastrectomy stratified by body mass index (BMI) category. Time is shown in months from surgery to death from any cause or censoring. Between-group differences were assessed using the log-rank test (*p* = 0.095).

During follow-up, long-term outcomes were unfavorable for the cohort as a whole, consistent with the substantial burden of advanced-stage disease. Kaplan–Meier analyses stratified by BMI did not demonstrate a statistically significant difference in OS across the four BMI categories (log-rank *p* = 0.095; Figure 1; Table 2). Underweight patients displayed the lowest survival probability (5-year OS 16.4%). The corresponding 5-year OS in normal-weight patients was 25.9%, while overweight and obese patients exhibited modestly higher estimates (27.4 and 29.3%, respectively); however, these between-group separations did not reach conventional thresholds for significance.

**Table 2:** OS by BMI categories.

BMI category	*n*	5-year OS rate (%)
Underweight (<18.5)	116	16.4%
Normal (18.5–22.9)	324	25.9%
Overweight (23.0–24.9)	117	27.4%
Obese (≥25.0)	311	29.3%
Log-rank test (among BMI categories)		*p* = 0.095

OS, overall survival. 5-year OS rates are Kaplan–Meier estimates of the probability of survival at 5 years after surgery. P values were calculated by the log-rank test.

**Figure 2:**
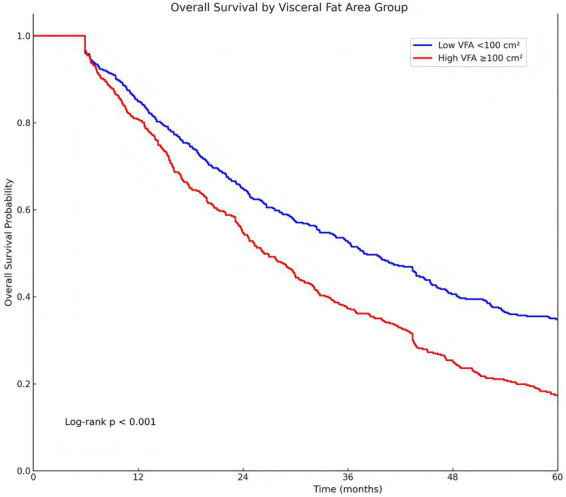
Overall survival (OS) by visceral fat area (VFA) group. Kaplan–Meier curves for OS stratified by computed tomography (CT)–derived VFA at the L3 level, categorized as low VFA (<100 cm^2^) versus high VFA (≥100 cm^2^). Time is shown in months from surgery to death from any cause or censoring. Survival distributions were compared using the log-rank test (*p* < 0.001).

By contrast, central adiposity—captured by VFA—was strongly related to survival. Individuals with high visceral fat (VFA ≥ 100 cm^2^) experienced significantly worse OS than those with low VFA (log-rank *p* < 0.001; Figure 2). Taken together, these unadjusted findings suggested that visceral adiposity carried adverse prognostic information, whereas BMI-defined obesity, as a categorical construct, did not show a clear relationship with OS.

### Multivariable analysis

To account for clinical confounding, a Cox proportional hazards model was constructed for OS incorporating BMI category, VFA, age, tumor stage, major postoperative complications, and adjuvant therapy (Table 3). After adjustment, BMI was not a robust independent prognostic marker except at the lower extreme. Underweight status (BMI < 18.5 kg/m^2^) was associated with inferior survival compared with normal BMI [adjusted hazard ratio (HR) 1.29, 95% confidence interval (CI) 1.03–1.62, *p* = 0.026]. In contrast, neither overweight (23.0–24.9 kg/m^2^) nor obesity (≥25.0 kg/m^2^) conveyed a statistically detectable survival advantage or penalty relative to normal BMI [HR 0.97 (0.77–1.23), *p* = 0.826; and HR 1.08 (0.91–1.29), *p* = 0.358, respectively].

**Table 3:** Multivariable cox regression for OS (*N* = 868).

Variable	Hazard ratio (HR) (95% CI)	*p*-value
BMI category (vs normal weight 18.5–22.9 kg/m^2^)		
– Underweight (<18.5)	1.29 (1.03–1.62)	0.026
– Overweight (23.0–24.9)	0.97 (0.77–1.23)	0.826
– Obese (≥25.0)	1.08 (0.91–1.29)	0.358
High VFA (≥100 cm^2^ vs. < 100 cm^2^)	1.56 (1.34–1.81)	< 0.001
Age (per 10-year increase)	1.09 (1.03–1.14)	0.001
Stage II (vs Stage I)	2.31 (1.88–2.83)	< 0.001
Stage III (vs Stage I)	4.71 (3.74–5.92)	< 0.001
Major postoperative complication (yes vs. no)	1.03 (0.66–1.62)	0.881
Adjuvant chemotherapy (yes vs. no)	1.12 (0.96–1.31)	0.155

Adjusted hazard ratios for BMI, VFA, age, stage, and complications (death events = 729).

HR, hazard ratio from a Cox proportional hazards model; CI, confidence interval. HR >1 indicates higher hazard (worse survival) and HR <1 indicates lower hazard (better survival). Reference categories: BMI 18.5–22.9 kg/m^2^ (normal), VFA <100 cm^2^ (low), Stage I, no major complication, and no chemotherapy. All variables listed were included simultaneously in the multivariable model.

**Figure 3:**
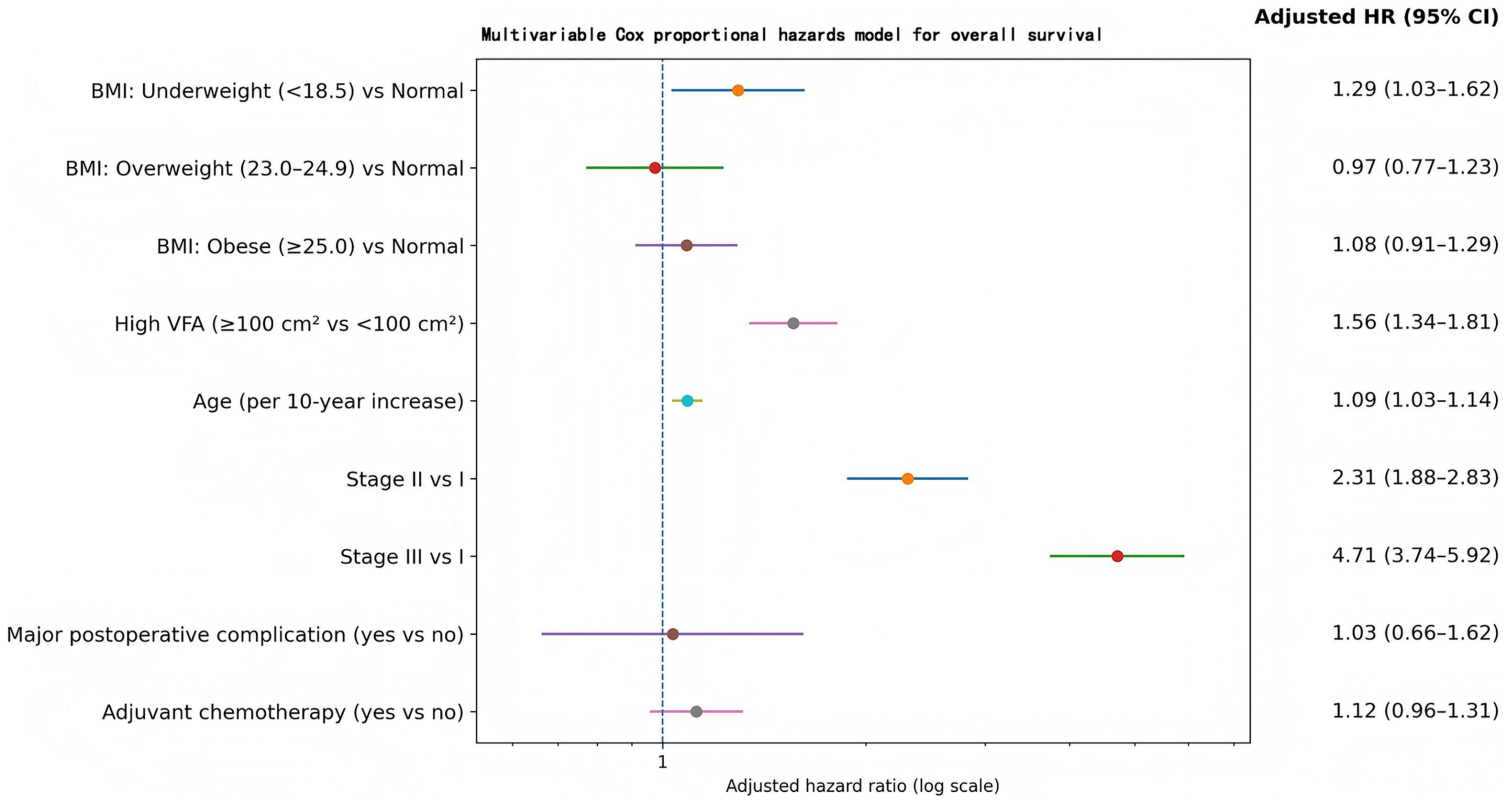
Multivariable Cox proportional hazards model for overall survival (OS). Forest plot of adjusted hazard ratios (HRs) with 95% confidence intervals (CIs) from the multivariable Cox regression model for OS. BMI categories are modeled with normal BMI as the reference; visceral adiposity is modeled as high VFA (≥100 cm^2^) versus low VFA (<100 cm^2^). Tumor stage (Stage II vs. I; Stage III vs. I), age (per 10-year) are included as covariates. The dashed vertical line indicates HR = 1.0 (no association), and the *x*-axis is displayed on a logarithmic scale.

VFA, however, retained a pronounced and independent association with mortality: high VFA (≥100 cm^2^) corresponded to an adjusted HR of 1.56 (95% CI 1.34–1.81, *p* < 0.001) compared with VFA < 100 cm^2^. Age also behaved as anticipated, with higher age predicting worse prognosis (HR 1.09 per 10-year increment, 95% CI 1.03–1.14, *p* = 0.001). Tumor stage exerted the largest effect sizes: relative to Stage I, Stage II carried an HR of 2.31 (1.88–2.83) and Stage III an HR of 4.71 (3.74–5.92), with both associations highly significant (*p* < 0.001). Major postoperative complications (Clavien–Dindo grade ≥III) were not independently associated with OS after covariate adjustment (HR 1.03, 95% CI 0.66–1.62, *p* = 0.881). Similarly, adjuvant chemotherapy did not reach statistical significance as an independent predictor in this specification (HR 1.12, 95% CI 0.96–1.31, *p* = 0.155). The forest plot (Figure 3) visually emphasizes this pattern: visceral adiposity and underweight BMI—alongside age and stage—were prognostic, whereas BMI ≥ 25 kg/m^2^ and major complications were not.

### Postoperative complications

**Figure 4:**
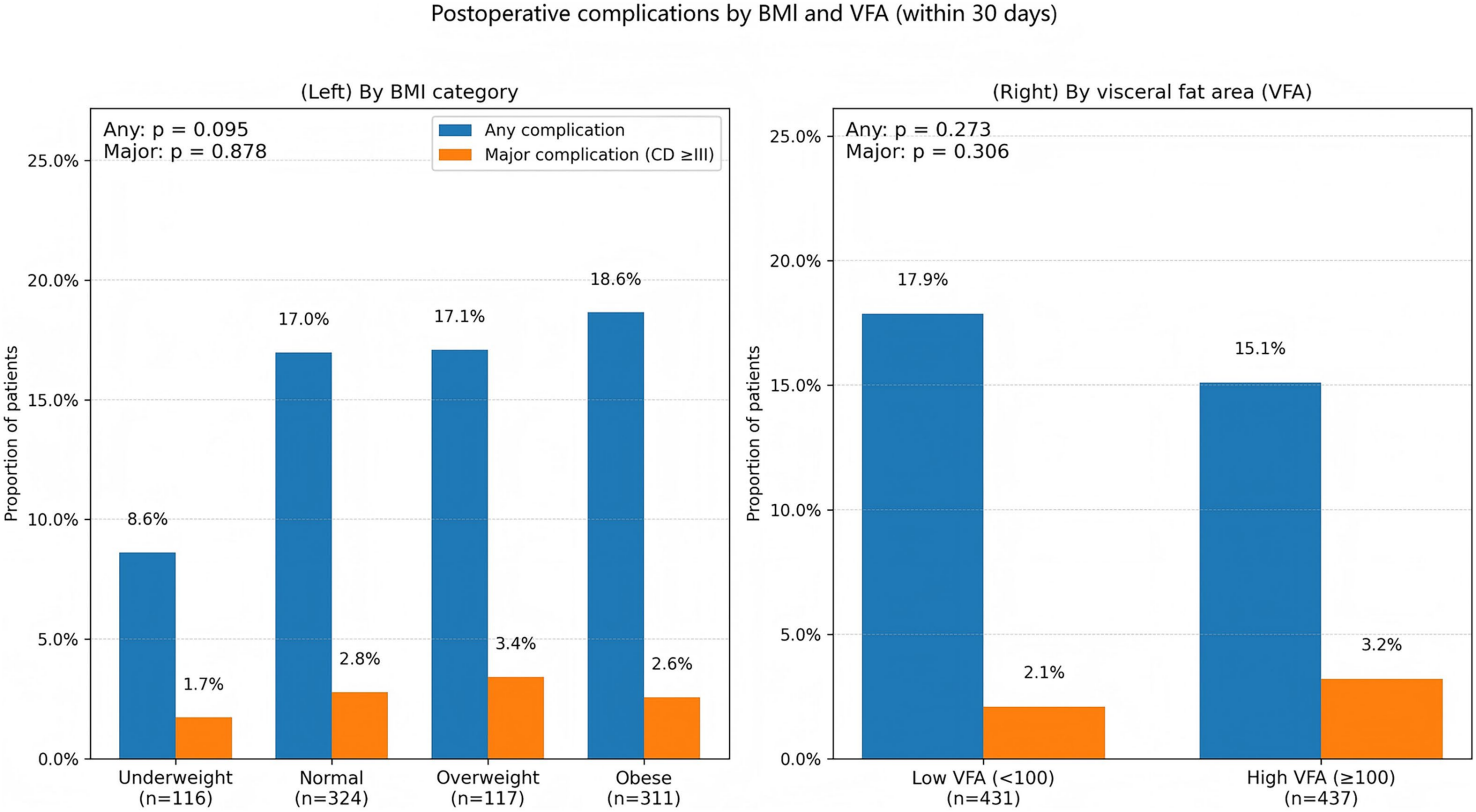
Postoperative complications by BMI and VFA. Bar plots showing the proportion of patients experiencing postoperative complications within 30 days after surgery, stratified by (left) BMI category and (right) VFA group. “Any complication” includes all recorded postoperative events; “major complication” denotes Clavien–Dindo grade ≥III. Group differences were evaluated using categorical tests as specified in the statistical analysis, with *p* values displayed on the plots (BMI: any complication *p* = 0.095; major complication *p* = 0.878; VFA: any complication *p* = 0.273; major complication *p* = 0.306).

Postoperative morbidity was evaluated across both BMI- and VFA-defined groups. Overall, 16.5% of patients developed at least one surgical complication, and 2.6% experienced a major complication (Clavien–Dindo ≥III). When stratified by BMI, the incidence of “any complication” showed no statistically significant difference among categories (*p* = 0.095; Table 4). Underweight patients showed the lowest rate (8.6%), while higher rates were observed in normal-weight patients (17.0%), whereas normal-weight, overweight, and obese patients had broadly similar rates (17.0, 17.1, and 18.6%, respectively; Figure 4). This distribution is consistent with a greater burden of minor postoperative issues among patients with higher BMI (e.g., wound-related events), though the data do not pinpoint mechanisms. Importantly, major complications were uncommon and did not differ by BMI (*p* = 0.878). In contrast to BMI, visceral adiposity was not associated with postoperative morbidity. High-VFA patients (≥100 cm^2^) had an “any complication” rate of 15.1% compared with 17.9% in the low-VFA group (*p* = 0.273; Table 5), and major complications occurred in 3.2% versus 2.1%, respectively (*p* = 0.306; Figure 4). Thus, in this cohort, VFA did not appear to track short-term postoperative risk, despite its strong relationship with long-term survival.

**Table 4:** Postoperative complications by BMI category.

Complication	Underweight (*n* = 116)	Normal (*n* = 324)	Overweight (*n* = 117)	Obese (*n* = 311)	*p*-value
Any complication	10 (8.6%)	55 (17.0%)	20 (17.1%)	58 (18.6%)	0.095
Major complication	2 (1.7%)	9 (2.8%)	4 (3.4%)	8 (2.6%)	0.878

Percentages are within each BMI subgroup. *P* values compare complication rates across BMI groups (chi-square tests; Fisher’s exact test used as appropriate for small counts). Major complication was defined as Clavien–Dindo grade ≥ III.

**Table 5:** Postoperative complications by VFA category.

Complication	Low VFA (<100 cm^2^, *n* = 431)	High VFA (≥100 cm^2^, *n* = 437)	*p*-value
Any complication	77 (17.9%)	66 (15.1%)	0.273
Major complication	9 (2.1%)	14 (3.2%)	0.306

Percentages are within each VFA subgroup. P values compare complication rates between VFA groups (chi-square tests; Fisher’s exact test used as appropriate for small counts). Major complication was defined as Clavien–Dindo grade ≥ III. BMI, body mass index; VFA, visceral fat area.

### Correlation between BMI and VFA

**Figure 5:**
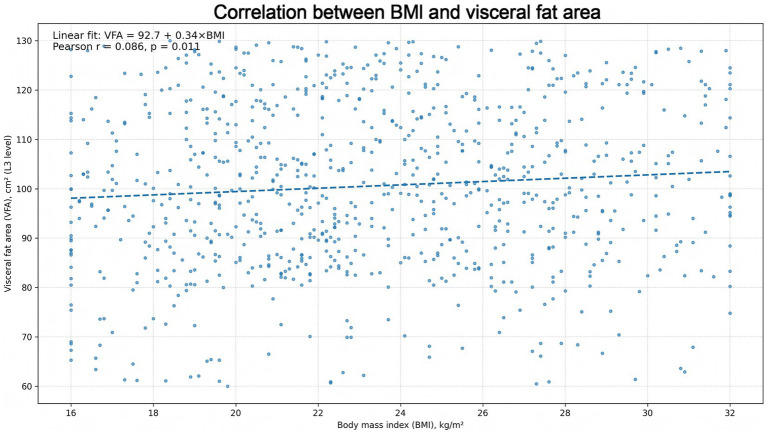
Correlation between BMI and VFA. Scatter plot depicting the relationship between BMI (kg/m^2^) and CT-derived VFA (cm^2^) measured at the L3 level. Each point represents one patient. The dashed line represents the fitted linear trend. The Pearson correlation coefficient is shown on the plot (*r* = 0.086), indicating a weak correlation between BMI and visceral adiposity in this cohort.

The relationship between BMI and VFA proved weak. As illustrated by the BMI–VFA scatter plot (Figure 5), wide inter-individual variability in visceral adiposity persisted at any given BMI: patients with relatively low BMI sometimes exhibited high VFA, and the reverse pattern was also evident. The Pearson correlation coefficient was modest (*r* = 0.086, *p* = 0.011), implying that BMI captured only a limited portion of central fat accumulation in this population. Operationally, BMI and VFA behaved as related but non-equivalent proxies for body composition, which plausibly helps reconcile the discordant prognostic signals observed for BMI categories versus VFA.

### Subgroup analysis by age

Median OS was 1,065 days (95% CI 942–1,235) among patients younger than 60 years, compared with 851 days (95% CI 747–974) among those aged ≥60 years. Estimated survival rates further reflected this separation: 3-year OS was 49.1% in the <60-year group versus 40.6% in the ≥60-year group, and 5-year OS was 31.0% versus 21.1%, respectively (Table 6).

**Table 6:** OS outcomes by age subgroup.

Outcome	<60 years	≥60 years	*p*-value
Median OS (days)	1,065	851	0.0005 (log-rank)
3-year OS (%)	49.1%	40.6%	—
5-year OS (%)	31.0%	21.1%	—

*OS, overall survival*.

In multivariable Cox regression incorporating BMI and VFA, age ≥60 years remained independently associated with higher mortality (HR 1.30, 95% CI 1.12–1.50; *p* < 0.001; Table 7). BMI did not show an independent association with OS when modeled continuously (per 1 kg/m^2^ increase: HR 0.985, 95% CI 0.968–1.003; *p* = 0.10). VFA, however, demonstrated a positive association with mortality risk (per 1 cm^2^ increase: HR 1.012, 95% CI 1.008–1.016; p < 0.001), reinforcing the prognostic relevance of visceral adiposity even after accounting for BMI.

**Table 7:** Multivariate cox regression for OS (adjusted for BMI and VFA).

Variable	HR	95% CI	*p*-value
Age ≥60 vs. < 60	1.30	1.12–1.50	<0.001
BMI (per 1 kg/m^2^)	0.985	0.968–1.003	0.10
VFA (per 1 cm^2^)	1.012	1.008–1.016	<0.001

HR, hazard ratio; CI, confidence interval; VFA, visceral fat area.

## Discussion

In this retrospective cohort of patients treated with curative-intent gastrectomy, the prognostic relevance of BMI was examined in parallel with imaging-defined visceral adiposity, with postoperative morbidity and long-term OS treated as distinct—though not necessarily independent—endpoints. Several inferences emerge from the analyses. First, increasing BMI, considered in conventional categories, did not translate into inferior OS, whereas underweight status was consistently accompanied by markedly worse survival, a pattern frequently interpreted under the rubric of an “obesity paradox” in gastric cancer. Second, once adiposity was interrogated by distribution rather than by total mass, central obesity—operationalized through VFA—carried adverse prognostic information that persisted after adjustment for tumor stage and other clinical covariates. Third, effect modification by age was evident for BMI, while the detrimental association of visceral adiposity with OS appeared comparatively stable across age strata. Collectively, the results argue—perhaps uncomfortably for BMI-centric risk assessment—that “weight” and “fat topology” are not interchangeable constructs when prognosis is being estimated in gastric cancer.

The observation that higher BMI was not aligned with poorer survival, juxtaposed against the pronounced vulnerability of underweight patients, is consonant with the expanding literature emphasizing nutritional and functional reserve as determinants of outcome after gastrectomy (15–17). Low BMI in this setting rarely signifies a benign constitutional phenotype; more often, it reflects cancer-associated cachexia, sarcopenia, and compromised immune competence, any of which can diminish tolerance to major surgery and attenuate the capacity to complete systemic therapy (16, 17). By contrast, modest excess body weight may confer metabolic “buffering”—greater energy stores and, in some patients, more preserved lean tissue—thereby moderating the physiologic shock of resection and subsequent treatments (17, 18). That interpretation accords with systematic reviews and meta-analyses suggesting that risk is concentrated at the lower tail of BMI, whereas overweight or mild obesity tends to yield neutral estimates once definitions and confounder adjustment are accounted for (15, 19). Consistent with this, recent prospective surgical evidence supports the diagnostic and prognostic value of structured malnutrition assessment tools, which could be considered alongside imaging-derived visceral fat measures in perioperative risk stratification (20).

Yet BMI, as an aggregate ratio, is blunt: it compresses heterogeneous body composition states into a single number, and it cannot discriminate lean mass from fat mass, nor subcutaneous from visceral compartments. When distribution was explicitly considered, visceral adiposity emerged as a more discriminating prognostic signal. High VFA was associated with significantly worse OS independent of BMI, tumor stage, and other measured covariates (21–25). Biologically, this is not a trivial association in search of a narrative. Visceral adipose tissue is metabolically active, capable of sustaining chronic low-grade inflammation, insulin resistance, and perturbed adipokine signaling—conditions that may plausibly facilitate tumor progression while simultaneously impairing host antitumor immunity (26, 27). In mechanistic terms, adipose-derived mediators can perpetuate systemic inflammatory tone and metabolic dysregulation, offering a coherent pathophysiologic scaffold for the adverse phenotype linked to visceral fat accumulation (28).

A notable nuance in the present cohort is that visceral adiposity did not materially elevate postoperative complication rates, despite its clear linkage to inferior long-term survival. Such dissociation is informative: it hints that the harms associated with visceral fat may operate predominantly through metabolic–oncologic pathways rather than through short-horizon surgical morbidity (26–28). The broader literature, admittedly, is not uniform on this point. Reports differ regarding whether visceral fat meaningfully increases postoperative complications, with heterogeneity likely driven by operative approach, patient selection, and definitional choices for morbidity endpoints (20–23). In laparoscopic cohorts, VFA has sometimes tracked operative difficulty and specific intraoperative adverse events more closely than BMI, although the direction and magnitude of associations with postoperative outcomes vary across datasets and complication taxonomies (21, 23, 29). It would be an overreach to treat “visceral fat” as a universal surrogate for perioperative risk without attending to these methodological contingencies.

Notably, prior studies have reported inconsistent associations between visceral fat and survival. Some systematic reviews and meta-analyses have even suggested that higher visceral fat may be associated with improved overall survival despite a higher incidence of postoperative complications, underscoring heterogeneity across cohorts and analytic approaches. For example, Matsui et al. found that high visceral fat increased severe complications yet was associated with improved long-term prognosis in advanced gastric cancer after propensity score matching (10). Such discrepancies may reflect differences in patient mix, definitions/cut-offs for visceral obesity, treatment patterns, and analytic adjustment (e.g., weight loss and muscle depletion). Therefore, our findings should be interpreted in the context of our cohort and warrant prospective multicenter validation.

In our study, underweight BMI predicted worse survival, BMI-defined overweight/obesity showed no clear survival penalty, and high CT-derived visceral adiposity independently predicted inferior OS. This pattern is consistent with critiques of BMI and the “obesity paradox,” where reverse causation and residual confounding (e.g., smoking and nutritional status) may bias BMI–survival associations; complementary body-composition and nutrition metrics may thus be needed alongside BMI (28–31).

In this context, the growing reliance on CT-derived body composition measures is more than a methodological fashion. Imaging-based phenotyping has repeatedly highlighted the prognostic relevance of visceral adiposity and muscle-related states. Gastric cancer–focused syntheses and cohorts increasingly support VFA as a candidate imaging biomarker for survival risk stratification (22, 24, 25). Equally, age-linked body composition phenotypes—particularly sarcopenic obesity—suggest that the combination of high visceral fat and diminished muscle reserve may represent a distinctly adverse state that BMI can conceal in plain sight (27, 28). A patient can “look” acceptable on BMI and still carry a high-risk metabolic and functional profile.

Age-stratified analyses in this study further complicate any simplistic BMI narrative. The neutral or apparently protective association of higher BMI was more evident in younger patients, while attenuation—or even reversal—was suggested in older individuals, consistent with the notion that age-related redistribution of fat and decline in muscle quality can materially alter what a given BMI “means” physiologically (27, 28). Visceral adiposity, however, retained its adverse association across age strata, implying a robustness that BMI lacks (22, 24, 25). This pattern coheres with geriatric nutrition guidance: BMI can misclassify risk in older adults because fat redistribution and muscle loss may occur without dramatic BMI shifts (32).

Clinically, several implications follow. Prognostic assessment and therapeutic decision-making should not lean on BMI in isolation. Elevated BMI should not be reflexively equated with poor outcomes, nor should treatment intensity be reduced solely on the basis of body weight. At the same time, the data support integrating CT-derived body composition measures—especially visceral fat quantification—into routine preoperative evaluation, given that these measures are typically retrievable from standard staging CT and can refine risk stratification beyond clinicopathologic variables. The broader management corollary is that nutritional and metabolic care should be individualized: aggressive weight loss is unlikely to be universally appropriate in gastric cancer, but targeted strategies aimed at reducing visceral adiposity, preserving skeletal muscle, and dampening systemic inflammation may be rational avenues for improving outcomes. Interventional work is therefore warranted, spanning perioperative nutritional optimization, exercise-based prehabilitation, and metabolic modulation. In that regard, a randomized trial demonstrating benefits from a personalized, multimodal, home-based prehabilitation program in gastric cancer surgery provides a pragmatic proof-of-concept for perioperative optimization approaches.

Several limitations constrain inference. The retrospective, single-center design invites selection bias and residual confounding despite multivariable adjustment. The cohort’s demographic composition—predominantly East Asian with comparatively modest BMI dispersion—may also restrict generalizability to Western settings where severe obesity is more prevalent. Moreover, the absence of direct skeletal muscle quantification, inflammatory biomarkers, and longitudinal body composition trajectories limits mechanistic interpretation and prevents evaluation of dynamic changes that may be prognostically salient. Finally, while follow-up permits assessment of mid-term survival, longer-horizon outcomes remain an open question.

Taken together, the study indicates that BMI and visceral adiposity are not redundant predictors in gastric cancer. Higher BMI was not associated with worse survival, and underweight status signaled a distinctly poor prognosis; yet excess visceral fat independently predicted inferior OS irrespective of age. The practical message is straightforward even if its implications are not: BMI, while convenient, is a partial and sometimes misleading proxy. Future prospective, multicenter studies that combine CT-based body composition profiling with mechanistic biomarkers are needed to sharpen risk stratification and to test interventions designed to modify the adverse metabolic and functional states associated with visceral adiposity.

## Conclusion

In this retrospective cohort of 868 patients undergoing curative-intent gastrectomy, BMI and CT-derived fat distribution conveyed non-equivalent prognostic information. Underweight status was independently associated with inferior OS, whereas overweight and obesity (by BMI categories) were not linked to increased mortality. In contrast, elevated visceral adiposity (VFA ≥ 100 cm^2^) emerged as a strong, independent predictor of worse survival, persisting after multivariable adjustment and appearing comparatively consistent across age strata. These findings support an “obesity paradox” framework for BMI while underscoring that central obesity—rather than body weight per se—better identifies patients at heightened long-term risk; incorporating routine CT-based visceral fat assessment into preoperative evaluation may therefore refine risk stratification and guide individualized perioperative management.

## Data Availability

The raw data supporting the conclusions of this article will be made available by the authors, without undue reservation.
